# Supplementary material for: Physiological and Transcriptional Responses of Industrial Rapeseed (Brassica napus) Seedlings to Drought and Salinity Stress
Source: Int J Mol Sci. 2019 Nov 9;20(22):5604. doi: 10.3390/ijms20225604 (PMC6888191; doi:10.3390/ijms20225604)
Supplement: Supplementary file 1 [file ijms-20-05604-s001.zip › Supplementary Materials/Figure S1. Venn diagram of DEGs.doc]

**Figure S1.** Venn Diagram of DEGs in the leaves and roots of *B. napus* plants subjected to drought or salinity stress. CK-L (CK, leaves) vs. D-L (Drought, leaves), CK-R (CK, roots) vs. D-R (Drought, roots), CK-L vs. S-L (salinity, leaves), CK-R vs. S-R (salinity, roots) represent DEGs under these two abiotic stresses
