# Supplementary material for: Physiological and Transcriptional Responses of Industrial Rapeseed (Brassica napus) Seedlings to Drought and Salinity Stress
Source: Int J Mol Sci. 2019 Nov 9;20(22):5604. doi: 10.3390/ijms20225604 (PMC6888191; doi:10.3390/ijms20225604)
Supplement: Supplementary file 1 [file ijms-20-05604-s001.zip › Supplementary Materials/Figure S2. Hierarchical clustering analysis of DEGs in each group based on log2 fold chang.doc]

**Figure S2.** Hierarchical clustering analysis of DEGs in each group based on log_2_ fold change. CK-L (CK, leaves) vs. D-L (drought, leaves), CK-R (CK, roots) vs. D-R (drought, roots), CK-L vs. S-L (salinity, leaves), CK-R vs. S-R (salinity, roots) represent DEGs under these two abiotic stresses. Red represents high expression level and blue represents low expression level. Each row represents a gene.The y-axis on the right indicates the fold change of DEGs.
