# Supplementary material for: Physiological and Transcriptional Responses of Industrial Rapeseed (Brassica napus) Seedlings to Drought and Salinity Stress
Source: Int J Mol Sci. 2019 Nov 9;20(22):5604. doi: 10.3390/ijms20225604 (PMC6888191; doi:10.3390/ijms20225604)
Supplement: Supplementary file 1 [file ijms-20-05604-s001.zip › Supplementary Materials/Figure S3. Phylogenetic trees MYBs and AP2-ERBPs families based on based on full-length predicted protein sequences..doc]

**Figure S3.** Phylogenetic trees MYBs and AP2-ERBPs families based on based on full-length predicted protein sequences. The phylogenetic trees were constructed using the conserved amino acid sequences by the neighbor-joining method implemented in MEGA software, version 4.1. The numbers at each branch point represent the bootstrap scores (1,000 replicates). Genes with red spot were reported TFs involved in regulation of expression of proline biosynthetic genes. MplMYB46 and MtMYBS1 are MYB members from *Medicago truncatula* [35] and *Betula platyphylla* [36], while JERF1 is a AP2-ERBP member from *Solanum lycopersicum* [38]. The other members in phylogenetic trees were differentially expressed TFs achieved from COGE database. (A) Phylogenetic tree of MYBs based on full-length predicted protein sequences. (B) Phylogenetic tree of AP2-ERBPs based on full-length predicted protein sequences.
