# Supplementary material for: Physiological and Transcriptional Responses of Industrial Rapeseed (Brassica napus) Seedlings to Drought and Salinity Stress
Source: Int J Mol Sci. 2019 Nov 9;20(22):5604. doi: 10.3390/ijms20225604 (PMC6888191; doi:10.3390/ijms20225604)
Supplement: Supplementary file 1 [file ijms-20-05604-s001.zip › Supplementary Materials/Figure S4. Analysis of putative cis-regulatory elements.doc]

**Figure S4.** Analysis of putative cis-regulatory elements in the promoters of *BnOAT*, *BnP5CS1*, and *BnP5CS2*. The 1000-bp sequences upstream of *BnOAT* (BnaA06g36140D), *BnP5CS1* (BnaA03g18760D, BnaA05g05760D, BnaC03g72600D, BnaC04g05620D), and *BnP5CS2* (BnaC04g55570D, BnaA04g03460D) containing transcription factor (TF) binding sites were analyzed with PLACE (Plant Cis-element Regulatory DNA Elements, http://www.dna.affrc.go.jp/PLACE/) and plantCARE (plant cis-acting regulatory element,	http://bioinformatics.psb.ugent.be/webtools/plantcare/html/). Predicted TF binding sites in the sense and antisense orientation are illustrated above and below the black lines, respectively.
